# Superacute onset of Guillain–Barré syndrome after elective spinal surgery: A case report and literature review

**DOI:** 10.1097/MD.0000000000037925

**Published:** 2024-05-03

**Authors:** Xinyu Zhang, Deshui Yu

**Affiliations:** a Department of Anesthesiology, The Second People’s Hospital of Yibin, Yibin, China; b Clinical Research and Translational Center, Second People’s Hospital of Yibin City-West China Yibin Hospital, Sichuan University, Yibin, China.

**Keywords:** case report, complications, Guillain–Barré syndrome, spinal surgery

## Abstract

**Rationale:**

Guillain–Barré syndrome (GBS) epitomizes an acute peripheral neuropathy hallmarked by an autoimmune retort directed at the myelin sheath enwrapping peripheral nerves. While it is widely acknowledged that a majority of GBS patients boast a history of antecedent infections, the documentation of postoperative GBS occurrences is progressively mounting. Drawing upon an exhaustive compendium of recent case reports, the disease’s inception spans a gamut from within 1 hour to 1.2 years.

**Patient concerns:**

At this juncture, we proffer a singular case: an instance involving a 51-year-old gentleman who underwent lumbar spine surgery, only to encounter immediate debilitation of limb and respiratory musculature.

**Diagnoses:**

Post elimination of variables linked to anesthetic agents, encephalon, and spinal cord pathologies, a potent suspicion of superacute GBS onset emerged.

**Interventions:**

Subsequent to immunoglobulin therapy, plasmapheresis, and adjunctive support, the patient’s ultimate demise became manifest.

**Outcomes:**

No progress was found to date.

**Lessons:**

Given GBS’s potential to instigate paralysis, respiratory collapse, and autonomic nervous system aberrations, alongside other pernicious sequelae, coupled with the exceptional rarity of the temporal onset in this particular instance, it undeniably proffers an imposing conundrum for anesthetists in the realm of differential diagnosis and therapeutic conduct. During the postoperative convalescence phase under anesthesia, should the patient evince deviant limb musculature vigor and compromised respiratory sinews, the prospect of GBS must not be consigned to oblivion. Precision in diagnosis conjoined with apt therapeutic measures could well be the harbinger of a divergent denouement for the afflicted patient.

## 1. Introduction

Guillain–Barré syndrome (GBS) stands as an acute or subacute paralytic affliction, underpinned by an autoimmune response. With the successful eradication of poliomyelitis, GBS has emerged as a prevalent instigator of acute flaccid paralysis on a global scale. The precise pathogenesis of GBS remains shrouded in ambiguity; nevertheless, numerous investigations posit that it unfolds as an immune-mediated reaction, distinctly localized in its impact, entailing both cellular and humoral immunological components.^[1]^ Typically, its onset is traced to instances following respiratory or gastrointestinal infections, predominantly aligning with agents such as *Campylobacter jejuni*, herpes simplex virus, Epstein–Barr virus, or cytomegalovirus. Notably, GBS can also be incited by pregnancy, surgical interventions, or even vaccinations.^[2]^ Characteristic clinical manifestations of GBS encompass advancing muscular enfeeblement, sensory aberrations, and dampened reflexes. Concurrently, ancillary symptoms might manifest, encompassing deficits in cranial nerves or disturbances in autonomic functions (inclusive of arrhythmias, elevated or diminished blood pressure, intestinal obstruction, and urinary retention).^[3]^ While instances of postoperative GBS remain infrequent, they can propel swift, life-threatening deterioration by undermining respiratory musculature. A consequential percentage, approximately 24%, of GBS patients necessitate mechanical ventilatory support. Additionally, mortality rates are as high as 12%, attributable to complications such as pneumonia, cardiac complications, and infarctions. Moreover, a notable proportion of patients, up to 20%, continue to grapple with neurological sequelae posttreatment. The recuperative trajectory in GBS ensues as a consistent and gradual process, extending up to a span of 2 years.^[4–7]^ Drawing from antecedent records, a minority of GBS incidents are precipitated and ascertained subsequent to surgical interventions, exhibiting varying onsets as delineated in Table 1. A broader spectrum of postoperative GBS scenarios has been chronicled, encompassing spheres such as transplantation, cardiac, and abdominal surgeries.^[20–22]^ Furthermore, isolated instances have surfaced following spinal surgeries (refer to Table 1). Due to the perceptible resemblance between complications ensuing from spinal surgery and the clinical manifestations of GBS, the diagnostic landscape for GBS becomes notably intricate.^[6]^ In this detailed exposition, we present an illustrative case of GBS, sequenced subsequent to elective spinal surgery, concurrently embarking on a comprehensive review of pertinent literature.

**Table 1:** Guillain–Barré syndrome after elective spinal surgery.

Order	Reference	Age/sex	Surgery	Time	Examination	Ventilator support	Treatment
1	Tu et al^[8]^	87/M	Decompression of C6-C7	2 wk	EMG lumbar puncture	Yes	Declined IVIG Plasmapheresis
2	Torregrossa et al^[9]^	76/M	L4-L5 microdiscectomy	Within 2 h	EMG, lumbar puncture	Yes	IVIG corticosteroid
3	Dowling et al^[10]^	53/F	L4-L5 right endoscopic discectomy, L	10 d	EMG	No	IVIG, antiganglioside antibodies
4	Aymen et al^[11]^	62/F	L3-L4 lumbar decompression and fusion	2 wk	EMG	Yes	IVIG
5	Chen et al^[12]^	57/M	Lumbar fusion at L3-S1	9 d	Lumbar puncture	Yes	IVIG methylprednisolone
6	Sahai et al^[13]^	52/M	L4eL5 decompression and fusion	17 d	EMG, lumbar puncture	No	IVIG
7	Boghani et al^[6]^	58/M	L4-L5 right-sided hemilaminotomy	Within 3 h	EMG lumbar puncture	Yes	IVIG, plasmapheresis
8	Boghani et al^[6]^	40/M	L3-L4 lumbar hemilaminectomy	Within 3 h	EMG lumbar puncture	No	IVIG, plasmapheresis
9	Battaglia et al^[14]^	73/F	Kyphoplasty	7 d	EMG	No	IVIG
10	Miscusi et al^[15]^	55/M	C6-C7 approach with laminoplasty	36 h	EMG lumbar puncture	No	IVIG glucocorticoids
11	Son et al^[16]^	50/M	Spinal canal decompression and spinal fusion	10 d	EMG	Yes	IVIG
12	Cheng et al^[17]^	59/F	T1-T3 laminectomy	6 h	EMG lumbar puncture	Yes	IVIG
13	Riebel et al^[18]^	62/F	T12-sacrum laminectomy, instrumentation	25 d	Not available	Not available	IVIG plasmapheresis corticosteriod
14	Stambough et al^[19]^	33/F	T5-L2 instrumentation	8 d	EMG	Yes	Corticosteriod

EMG = electromyography, F = female, IVIG = intravenous immunoglobulin, M = male.

## 2. Case presentation

Written informed consent was duly acquired from the patient for the purpose of publishing this case report. A male patient, aged 51, presented with persistent lower back pain and radiating discomfort in both lower limbs, manifesting without an identifiable antecedent cause 2 years prior to admission. Although the pain abated while reclining, its recent escalation prompted an MRI investigation, which unveiled varying degrees of desiccation and degeneration in the intervertebral discs spanning T12–S1. The L5–S1 disc exhibited posterior protrusion toward the right. Concurrently, degenerative osseous overgrowth was discerned in the L3 and L4 conus. The patient refuted any history indicative of respiratory, circulatory, digestive, endocrine, or immune disorders. Likewise, there were no prior instances of surgery or trauma, nor recent records of vaccination. Muscular strength across all 4 limbs was within the normal spectrum, accompanied by physiologically sound reflexes and an absence of pathologic reflex responses. Prior to anesthesia administration, routine monitoring protocols encompassing pulse oximetry, electrocardiography, and noninvasive arterial pressure were executed. Baseline metrics, including heart rates, systolic and diastolic blood pressures, and mean arterial pressures, were documented pre-anesthesia. The total duration of the surgical procedure amounted to 3.8 hours, during which an infusion volume totaling 2500 mL was administered, encompassing 1100 mL of compound sodium chloride injection and 1500 mL of polygeline injection. The operative intervention comprised a posterior approach decompression featuring L5–S1 laminectomy, intervertebral disc nucleotomy, exploration and liberation of adhered nerve roots, coupled with interbody fusion involving iliac bone grafting. The surgical course transpired without notable impediments, accompanied by a blood loss of approximately 400 mL. The patient’s respiratory and circulatory functions sustained stability throughout the procedure. Post-procedure, the patient was administered antagonists to counteract the effects of benzodiazepine drugs and muscle relaxants. While the patient was responsive to verbal prompts within 5 minutes of extubation (intubation status), evidencing weak spontaneous respiration, low tidal volume, and generalized limb weakness, the medical team persisted with mechanical ventilation. After 20 minutes, the muscle strength in both lower limbs abruptly declined to grade 1, subsequently deteriorating further. Following this, the patient promptly experienced loss of consciousness, with both pupils dilated to approximately 6 mm, accompanied by a lack of light reflex. Autonomous respiration ceased, while heart rate, rhythm, and blood pressure exhibited no significant anomalies. In the aftermath of consultations with the neurosurgery and neurology departments, an urgent CT scan was conducted.

**Figure 1.**
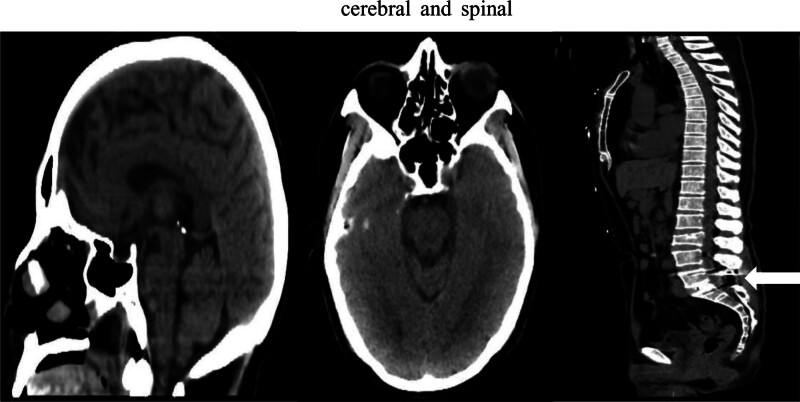
Cerebral and spinal.

Upon transfer to the Intensive Care Unit (ICU), the patient regained consciousness, demonstrating the capacity to furnish articulate and coherent responses. Absence of facial asymmetry was noted, and the pupils presented as uniform in size, measuring approximately 3 mm in diameter. Nevertheless, the patient’s muscular vigor in all 4 limbs had markedly waned when juxtaposed with preoperative baseline values, with the strength in both bilateral lower limbs subsiding to grade 1, and that in the upper limbs diminishing to grade 4. This enfeeblement exhibited a more pronounced effect on proximal musculature in comparison to distal counterparts. The patient proved incapable of maintaining satisfactory oxygen saturation through spontaneous respiration, necessitating continuous mechanical ventilation. Noteworthy was the substantial reduction in blood pressure from prior levels, culminating in the administration of a dopamine infusion to uphold hemodynamic stability. An urgent cerebral and spinal CT scan yielded results bereft of significant abnormalities (refer to Fig. 1). His complete blood count, infection indicators, serum electrolyte levels, liver function tests, and renal function were all normal. Following exhaustive multidisciplinary deliberation, a robust suspicion of GBS manifested. The therapeutic approach embraced intravenous immunoglobulin (IVIG) therapy, fresh frozen plasma, hormonal interventions, and nutritive reinforcement geared toward bolstering the nervous system. Although advocating cerebrospinal fluid (CSF) analysis was pertinent, the prospect of perturbing the accuracy of test outcomes and potentially inciting postoperative infection precluded lumbar spine surgery-induced CSF puncture. On the second postoperative day, electromyography and evoked potential assessments unveiled an absence of aberrations in nerve conduction across both upper and lower limbs. Progressively, by the third postoperative day, and consequent to the interventions enumerated earlier, the patient’s muscular potency exhibited gradual enhancement. Specifically, the muscle strength in the upper limbs surged to grade 4, while the lower limbs ameliorated to grade 1+. Due to economic reasons, the patient was relocated to an alternate medical facility after a fortnight. Tracking the case, the hospital performed a lumbar puncture, and the CSF examination revealed a pressure of 168 mmH2O, an absence of white blood cells, protein levels (1.60 g/L), glucose (4 mmol/L), and chloride (116.80 mmol/L).

We believe that the patient can be diagnosed with GBS. However, the patient refused further treatment and eventually died due to multiple organ failure.

## 3. Discussion

This particular patient presented an absence of antecedent infection or vaccination events preceding the surgical intervention. Previous data have demonstrated that nearly two-thirds of patients exhibit a history of antecedent ailments, predominantly manifesting as upper respiratory or gastrointestinal infections. However, the precise etiological agents underpinning these antecedent infections often prove elusive to ascertain.^[23]^ Classic GBS can manifest at any chronological phase, bereft of discernible seasonal fluctuations, with a slightly elevated incidence in males relative to females. Preeminent among the pathogens intricately linked with GBS is *C jejuni*, notably associated with the axonal manifestation of GBS.^[24]^ Multiple investigations have indicated the isolation of *C jejuni* from the fecal specimens of select patients, while the infection prevalence markedly diminishes within control cohorts. Although infection incidence displays consistency across diverse geographical domains, the alignment between infection and clinical phenotypes introduces variation.^[25]^ In recent times, mounting reports have emerged concerning postoperative GBS, thrusting postoperative and post-traumatic GBS into heightened prominence. An intriguing speculation beckons: could trauma (inclusive of surgery, fractures, miscarriages, cerebral hemorrhages, etc) function as an autonomous risk factor for GBS^[2,26,27]^? Presently, ongoing inquiry seeks to elucidate the potential for trauma to serve as a predictive parameter for GBS.

GBS can impact nerves spanning from the nerve root to the neuromuscular junction, with a primary involvement noted in the proximal nerve roots, spinal nerves, and posterior cranial nerves. Severe inflammatory reactions can incite secondary axonal degeneration, a phenomenon evidenced by ultrastructural investigations attributing a pivotal role to monocytes in the demyelinating cascade.^[28]^ The most prevalently embraced theory posits that neurological damage materializes consequent to an autoimmune process incited by infectious agents or alternative immunostimulatory triggers.^[29]^ However, an unforeseen observation has arisen, wherein instances of GBS have surfaced within severely immunocompromised patients. This has manifested notably in recipients of liver, kidney, heart, and lung transplants.^[20,30]^ The exact relationship between surgery and the heightened susceptibility to GBS remains enigmatic. A single-center study has proposed that nontraumatic postoperative neuropathy arises within a 30-day window post-surgery in patients who subsequently develop postoperative GBS, with 21 cases unveiling inflammatory alterations.^[31]^ Concerning postoperative GBS, a plausible mechanism postulated involves the release of specific antigens during surgical intervention, which in turn sparks autoimmune assaults directed at the nervous system.^[32]^ Furthermore, surgery engenders a disturbance in the equilibrium among constituents of the immune system, accentuated by an escalated production of immunoglobulins coupled with the depletion of lymphocytes; this concurrent perturbation can potentially serve as a precipitating factor for GBS.^[33]^ Certain researchers contend that transient immune suppression ensues during surgical procedures, thereby activating neuroendocrine pathways. The resultant immune suppression may pave the way for subclinical infections, ultimately prompting the production of cross-reactive antibodies, thereby contributing to the onset of GBS symptoms.^[2]^ Given the rarity of GBS and the intricacies entailed in its diagnostic process, extensive investigation remains imperative to fathom its intricate pathogenesis.

The cardinal clinical features characterizing classic GBS encompass motor paralysis bereft of reflexes, accompanied by potential sensory impairment. The trajectory of paralysis is notably ascending, commencing with the lower extremities and subsequently extending to encompass the upper limbs and facial musculature. In instances of pronounced severity, respiratory muscle debilitation and disruptions in autonomic functions might manifest. Gradual progression of weakness spans across several weeks, ultimately plateauing around the 4-week mark.^[2]^ Diagnosis is relatively straightforward, facilitated by hallmark symptoms and clinical indicators, CSF analysis, and electrophysiological assessments.

The onset of postoperative GBS may transpire anytime within the span of an hour to a year post-surgery. Instances of acute-onset GBS, immediately subsequent to anesthesia, have been documented, which can inadvertently mask GBS manifestations during the post-anesthesia recuperation period. Timely diagnosis and appropriate management of acute-onset postoperative GBS can pose a challenge for surgeons.

Management of GBS should encompass vigilant respiratory and circulatory support while maintaining a state of diligent monitoring. Successful prognosis pivots on averting severe complications such as respiratory failure and autonomic dysregulation. Presently, plasma exchange and high-dose IVIG therapy stand as the preferred interventions for severe GBS cases.^[34]^ Notwithstanding, the administration of corticosteroids remains embroiled in ongoing debate. In certain underdeveloped regions where plasma exchange and IVIG are not readily accessible, high-dose corticosteroid regimens for GBS patients might yield limited benefits in acute scenarios.^[35]^ Recognizing the malady at an early juncture and initiating prompt therapeutic measures play pivotal roles in shaping patient prognosis.

GBS has garnered global attention.^[1]^ Drawing from North American and European demographics, the prevalence of GBS spans a range of 0.81 to 1.91 cases per 100,000 person-years. Notably, each decade of age advancement corresponds with a 20% escalation in the incidence rate. In a comprehensive IGOS study involving a cohort of over 900 GBS patients, the median age approximated 51 years, with the bulk of cases clustered within the 50 to 69 years age bracket.^[36]^ The landscape in Japan reflects an incidence rate of 0.44 cases per 100,000 person-years.^[37]^ While in China, this stands at around 0.67 cases per 100,000 person-years.^[38]^ Bangladesh portrays an incidence rate of 1.5 to 2.5 cases per 100,000 person-years among adults and 3.25 cases per 100,000 person-years among children. Parallel to Europe and the United States, the Middle East registers a GBS incidence comparable to these regions.^[39]^ As healthcare standards advance across various geographic areas, the overarching incidence of GBS witnesses a surge.

To encapsulate, GBS is relinquishing its rarity, particularly underscored by the surge in reports of postoperative GBS cases. Predominantly, the surge in occurrences is observable in patients undergoing orthopedic spine surgery, as summarized in Table 1 in recent times. In the aftermath of spine surgery, when patients manifest muscle debility or sensory perturbations, a meticulous diagnostic process that delves into meticulous differentiation becomes imperative. Anesthesiologists and surgeons alike must embark on a comprehensive exploration to rule out potential culprits such as surgical errors, toxic or metabolic factors, and primary neurological pathologies. In instances where conventional imaging modalities and laboratory assessments prove inadequate to elucidate limb weakness, sensory irregularities, and autonomic disruptions, GBS warrants inclusion within the differential diagnosis. An infrequent occurrence entails rapid symptom progression within hours post-surgery, presenting an imminent life-threatening risk for the patient.

## 4. Conclusion

In this particular instance, the patient exhibited immediate postoperative symptoms, which swiftly escalated into severe manifestations, notably encompassing respiratory failure. This trajectory diverged markedly from the conventional presentation of GBS. Initially, the medical team did not entertain the notion of GBS as a plausible diagnosis, leading to a subsequent lapse in the initiation of plasma exchange and immunoglobulin therapy. Despite GBS standing as a relatively infrequent sequel to spine surgery, any form of delay in both diagnosis and therapeutic intervention could culminate in calamitous consequences, potentially proving fatal. Hence, we posit that augmentation of healthcare practitioners’ cognizance concerning postoperative GBS remains imperative, further underscored by the crucial significance attributed to discerning atypical GBS presentations. Such vigilance can optimally facilitate the early and precise diagnosis of GBS, thereby optimizing clinical outcomes for the afflicted patients.

## Author contributions

**Conceptualization:** Xinyu Zhang.

**Data curation:** Xinyu Zhang.

**Formal analysis:** Xinyu Zhang.

**Funding acquisition:** Deshui Yu.

**Investigation:** Xinyu Zhang.

**Methodology:** Deshui Yu.

**Project administration:** Xinyu Zhang.

**Resources:** Xinyu Zhang.

**Software:** Xinyu Zhang.

**Supervision:** Deshui Yu.

**Validation:** Deshui Yu.

**Visualization:** Xinyu Zhang.

**Writing—original draft:** Xinyu Zhang.

**Writing—review & editing:** Deshui Yu.
